# Pharmacokinetics of Micafungin in Critically Ill Patients

**DOI:** 10.1038/s41598-019-53093-6

**Published:** 2019-11-28

**Authors:** Silke Gastine, Christian Lanckohr, Magalie Blessou, Dagmar Horn, Manfred Fobker, Daniela Bause, Georg Hempel, Björn Ellger

**Affiliations:** 1 Institute of Pharmaceutical and Medical Chemistry – Department of Clinical Pharmacy, Westfälische Wilhelms-Universität Münster, Münster, Germany; 2 Department of Anesthesiology, Intensive Care and Pain Medicine, University Hospital Münster, Münster, Germany; 3 Department of Pharmacy, University Hospital Münster, Münster, Germany; 4 Center for Laboratory Medicine, University Hospital Münster, Münster, Germany; 5 Department of Anesthesiology, Intensive Care Medicine and Pain Therapy, Klinikum Westfalen, Dortmund, Germany

**Keywords:** Computational models, Fungal infection

## Abstract

We investigated covariates of pharmacokinetics of micafungin in critically ill patients. After application of micafungin, plasma samples were collected. Non-linear mixed effects modelling (NONMEM 7.3) was used to develop the pharmacokinetic model. Using this model, the adequacy of a fixed 100 mg dosing regimen was evaluated in the study cohort. A two-compartment model with linear elimination was found to describe the obtained data. SOFA score was identified as a significant covariate on both clearance and central volume of distribution, respectively. Patients in highly critical condition, represented by a SOFA above 10 showed a 30.8% lower central volume of distribution than the less critically ill patients. For patients with bilirubin levels above 4 mg/dl, clearance was decreased by 21.1%. Renal replacement therapy (RRT) did not influence micafungin clearance or the volumes of distribution. In a posthoc evaluation of the modeled population, 100 mg micafungin was suitable when assessing the PKPD targets (AUC/MIC) for *C*. *albicans* and *C*. *glabrata*, with insufficient target attainment for *C*. *parapsilosis*. Micafungin pharmacokinetics appear not to be influenced by the status of RRT. A dose of 100 mg micafungin is suitable for infections with *C*. *albicans* and *C*. *glabrata* in critically ill patients.

## Introduction

Invasive fungal infections in critically ill patients are a serious threat and a cause of high mortality. In unselected patient cohorts, fungi account for 10–20% of infections in the intensive care unit. *Candida* species are the predominant fungi isolated in critically ill surgical patients^1,2^. A typical focus of infection in this collective is the intraabdominal space, where *Candida* peritonitis and candidemia develop after perforation of a hollow viscus^3^. Besides this typical mechanism, candidemia can also occur as a device-related infection, e.g. by inoculation through an intravascular catheter^4,5^.

The contemporary treatment of invasive candidiasis and candidemia relies on echinocandines as the primary choice of antifungal agents. This strategy is recommended by several clinical guidelines dealing with invasive fungal infections^6–8^. After caspofungin and anidulafungin, micafungin was the third echinocandin antifungal agent approved by the European Medicines Agency in 2008. It is licensed for the treatment of all types of invasive candidiasis, esophageal candidiasis and for prophylaxis in patients undergoing stem cell transplantation^9^.

Micafungin is available as an intravenous formulation, which is typically infused over 1 h. It is characterized by potent antifungal activity, with few drug interactions and no relevant toxicity. The mechanism of action of micafungin is the inhibition of fungal β-(1, 3) glucan synthase complex, leading to the depletion of cell-wall glucan and consecutive osmotic instability causing cell death^9^.

Echinocandins exhibit concentration-dependent killing of *Candida* species. Therefore, the 24-h area under the concentration time curve divided by the minimal inhibitory concentration (AUC/MIC) is the pharmacodynamic parameter that best describes the dose-response relation of this drug^10^. Micafungin has a small volume of distribution (~119 ml/kg) and the drug is highly protein bound (>99%). The primary binding protein is albumin. Linear pharmacokinetics were observed for daily doses between 12.5 mg and 200 mg as well as 3 mg/kg and 8 mg/kg^9^. The pharmacokinetics and pharmacodynamics of micafungin have recently been described by Wasmann *et al*. in detail^11^.

Regarding special populations, Herbert *et al*. did not find an effect of altered renal dysfunction on micafungin pharmacokinetics in a non-compartmental analysis^12^. In patients with hepatic dysfunction, micafungin AUC was lower than in control subjects, other pharmacokinetics parameters such as weight adjusted clearance or volume of distribution did not show a statistical difference^12^. According to Undre *et al*., AUC is significantly lower, and clearance was found to be higher in patients with severe hepatic dysfunction, which might be secondary to reduced plasma albumin levels in these subjects^13^. An increase in free drug levels during hypoalbuminemia is thought to cause an increased clearance in these patients.

No dose adjustment is necessary for elderly patients, as exposure and disposition of micafungin in healthy subjects aged 66–78 years were not significantly different from those aged 20–24 years^9^.

Previous pharmacokinetic analyses indicate a body weight-dependent clearance of micafungin. There was no relationship between weight and clearance in subjects between 43 kg and 66 kg, but the clearance increased with a function of weight, beyond a threshold of 66 kg. It remains to be determined, whether this finding suggests that subject weighing more than 66 kg might fail to achieve optimal AUC/MIC ratios with standard dosages^14,15^.

Critically ill patients in the intensive care unit (ICU) are a heterogeneous cohort of patients. Various degrees of organ dysfunction, changes in volume status and metabolic imbalances signify critical illness. Taken together, these physiologic abnormalities have considerable influences on the pharmacology of many substances. Regarding antiinfectives, these changes can have deleterious consequences. If the exposure to an antibiotic or antimycotic compound is too low, the efficacy is compromised, subsequently raising the potential of therapeutic failure^16^.

The objective of this pharmacokinetic study was to assess the pharmacokinetics of micafungin in a mixed cohort of critically ill adult patients with diverse organ failures, including renal failure supported by renal replacement therapy (RRT). Furthermore, the impact of different modes of RRT on pharmacokinetics was explored. Blood levels were then correlated with AUC/MIC of different candida species to evaluate pharmacologic adequacy of a daily dose of 100 mg micafungin.

## Results

This study of micafungin pharmacokinetics included 36 patients (24 male and 12 female) aged between 22 and 84 years. The median weight was 94.5 kg (49.4 kg–162 kg).

All patients included were critically ill, as reflected by high compound scores for critical illness (APACHE 2, SOFA, SAPS 2). The majority of patients fulfilled sepsis criteria (SEPSIS-II), received vasopressors for hemodynamic support and were on mechanical ventilation. 23 patients received renal replacement therapy (RRT) for at least one day of the study while the remaining 13 patients never received RRT. A summary of patient characteristics and covariates is shown in Table 1.

**Table 1:** Patient characteristics (Abbreviations: BMI: body mass index; ALT: alanine-aminotransaminase; AST: aspartate-aminotransaminase; BCHE: buturylcholine esterase; APACHE 2: acute physiology and chronic health evaluation 2; SOFA: sequential organ failure assessment; SAPS2: simplified acute physiology score 2; CVVHDF: continuous veno-venous hemodiafiltration; SLEDD: slow low-efficiency daily dialysis; ECMO: extracorporal membrane oxygenation).

Characteristic	Value
Age [years], median (range)	65 (22–84)
Weight [kg], median (range)	94.5 (49.9–162)
BMI [kg/m^2^], median (range)	29.87 (18.15–93.66)
Albumin [g/dL], median (range)	2.5 (1.5–4.2)
Plasma protein [g/dL], median (range)	5.3 (2.5–7.3)
ALT [units/L], median (range)	53 (8–4615)
AST [units/L], median (range)	110 (18–12550)
BCHE [units/L], median (range)	2214 (509–6357)
Creatinine [mg/dL], median (range)	1.1 (0.3–6.2)
Creatinine Clearance [ml/min], median (range)	97 (23–286)
APACHE 2, median (range)	35 (13–46)
SOFA, median (range)	12 (2–22)
SAPS2, median (range)	55 (14–104)
Sepsis	78% (28/36)
Vasopressors	75% (27/36)
Mechanical Ventilation	86% (31/36)
Renal replacement therapy	64% (23/36)
CVVHDF	13
SLEDD	3
Intermittent Hemodialysis	5
CVVHDF and SLEDD	2
ECMO support (either venovenous or venoarterial)	16% (6/36)

Micafungin was started empirically in 25 patients, where the suspected foci of infection were either intraabdominally or device-associated (intravenous catheters, ECMO). In 11 patients the prescription was made to treat proven infections. Of those 11 patients, 8 had intraabdominal infections with candida species in intraoperative samples, two had candidemia with suspected infection of venous catheters and one had osteomyelitis of the sternum and mediastinitis after heart surgery. *Candida albicans* was isolated in 8 cases, *Candida glabrata* in 3 cases. The median length of micafungin therapy was 10,5 days (ranges 2–79 days) and treatment was continued for as long as treating physicians felt it was adequate. 15 patients died in the ICU; the other patients were discharged alive.

**Figure 1:**
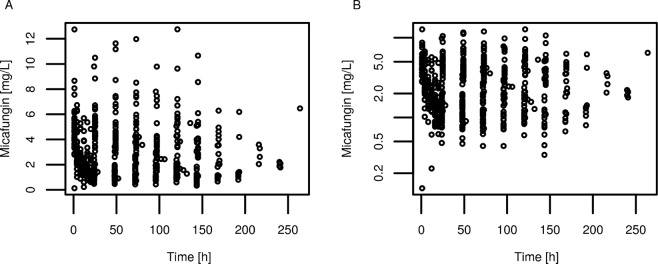
Measured plasma concentration vs. time profile – recorded as time after first dose. (**A**) Plasma concentrations shown in linear scale and (**B**) shown in logarithmic scale.

**Figure 2:**
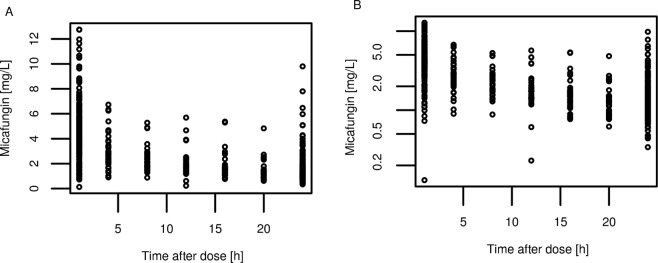
Measured plasma concentration vs. time profile - recorded as time after last dose. (**A**) Plasma concentrations shown in linear scale and (**B**) shown in logarithmic scale.

The measured plasma concentration plotted against time after first dose or time after last dose are shown in Figs 1 and 2, respectively. Samples were available at various time points across the treatment period, giving the opportunity to study micafungin pharmacokinetics during up to 10 days of treatment. No accumulation of micafungin was observed and a marked heterogeneity of drug concentrations in the critically ill patient population is clearly visible (Figs 1, 2).

### Population pharmacokinetic modelling

The basic structural model consisted of two compartments with a proportional error model. Clearance was tested as linear, nonlinear *Michaelis-Menten* type and as combined linear- nonlinear type. Out of these, the linear clearance best described the data. Interindividual variability (IIV) was allowed for clearance, central and peripheral volume of distribution. For these variabilities, a full covariance matrix was estimated.

Covariate testing was performed using stepwise covariate modelling (SCM) in PsN with a 5% forward inclusion and 1% backward elimination criterion. Through SCM, a significant covariate effect was shown for the SOFA score with a linear decrease in clearance with a factor of 0.024 per score point.

Plots of the parameters against covariates showed additional relations for SOFA on central volume of distribution, calculated as categorical effect for critically ill patients with a SOFA >10, resulting in a 30.8% lower central volume of distribution in the critically ill group.

In addition, a categorical effect for bilirubin on clearance with a cut-off for bilirubin >4 mg/dl was included, showing a 21.1% lower clearance in the group with elevated bilirubin levels. Parameter estimates for the final model are listed in Table 2.

**Table 2:** Final population pharmacokinetic model estimates (Abbreviation: RSE: residuals standard error).

Parameter	Empirical Bayes Estimate	RSE (%)
Clearance [L/h]	1.56	4.8
Central volume of distribution (L)	16.2	12.7
Inter-compartmental clearance [L/h]	14.4	7.7
Peripheral volume of distribution [L]	13.8	15.7
Proportional residual Error (%)	0.257	7.4
Inter-individual Variability Clearance (%)	25.9	9.8
Inter-individual Variability Peripheral volume of distribution (%)	70	13
Inter-individual Variability Central volume of distribution (%)	48.9	13.4

**Figure 3:**
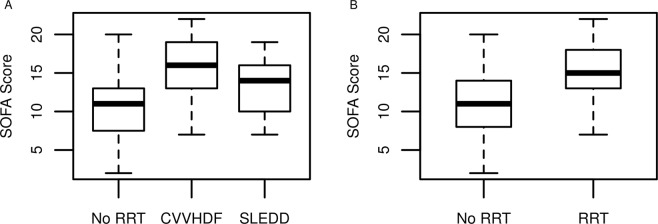
Correlation of SOFA score and renal replacement therapy differentiated by modality (**A**) and overall provision of RRT. RRT (renal replacement therapy), CVVHDF (continuous veno-venous hemodiafiltration), SLEDD (slow low-efficient daily dialysis).

All other covariates did not show a significant influence on any of the PK parameters. An influence of the patients’ body weight, especially with obese patients, as reported by Hall *et al*.^15^ was not detected. Renal replacement therapy modalities (CVVHDF, SLEDD) were correlated with SOFA scores, indicating that highly critically ill patients tend to have a higher chance of receiving renal replacement therapy at any time during treatment and more CVVHDF treatments are recorded in this group. The visual exploration of the differences in SOFA distribution for distinct modalities of RRT is shown in Fig. 3. During the study, patients could switch between the modalities as clinically necessary and by decision of the treating physicians. This somewhat random shift makes a classical correlation analysis difficult. A plain analysis of differences of the groups’ mean SOFA scores by t-test showed differences (p > 0.01) between mean SOFA scores across all treatment groups (no RRT, CVVHDF, SLEDD). The impact of disease state (i.e. critical illness reflected by SOFA score) and RRT group were tested independently on the estimated population pharmacokinetic parameters, revealing only an influence of the SOFA score as a relevant covariate.

**Figure 4:**
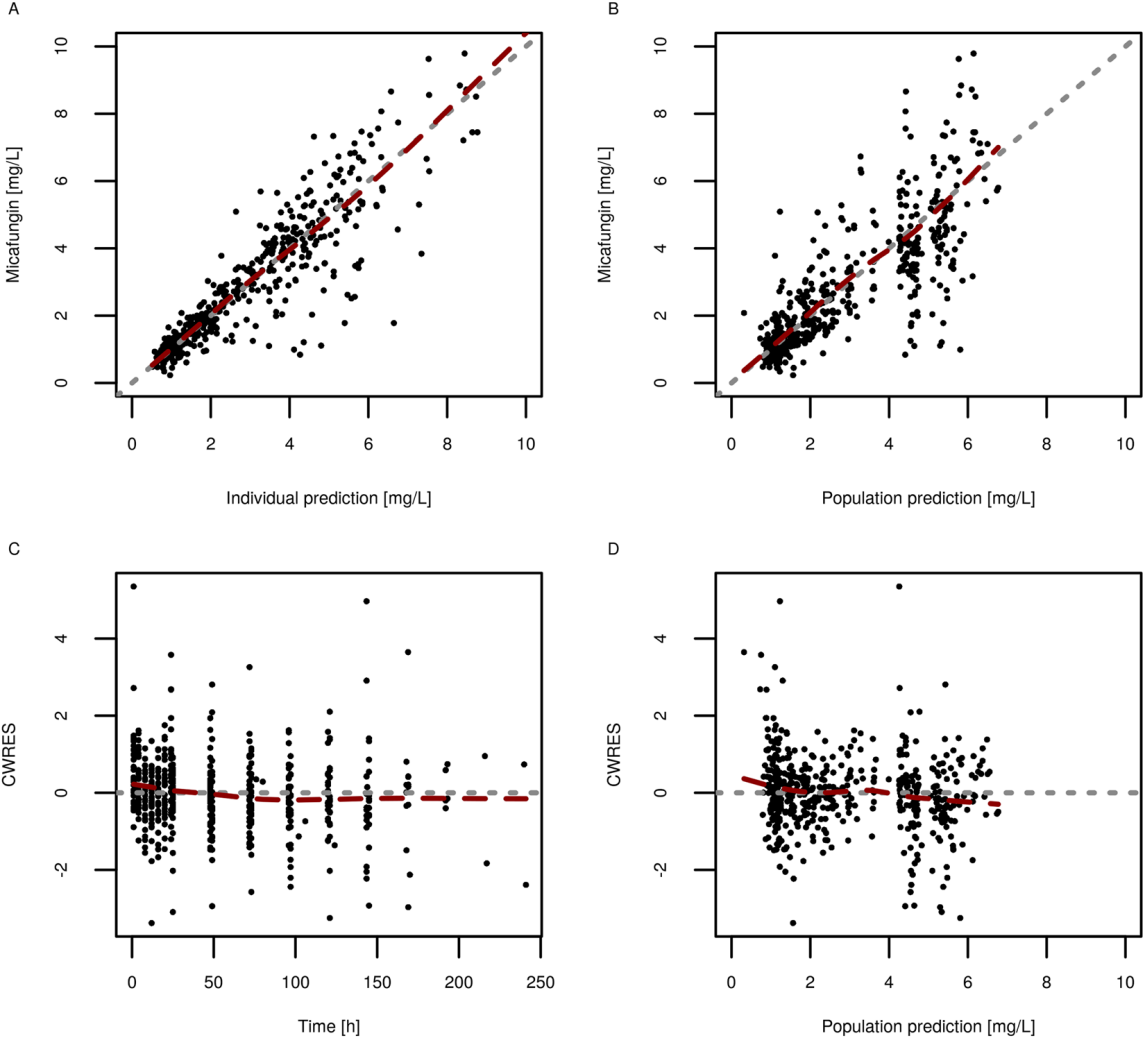
Goodness of Fit Plot for individual (**A**) and population **(B**) predictions and conditional-Weighted-Residuals (CWRES) plotted against Time (**C**) and populations predictions (**D**); black dots, observed/ predicted values; dashed line, line of identity; red line, Loess-Fit.

**Figure 5:**
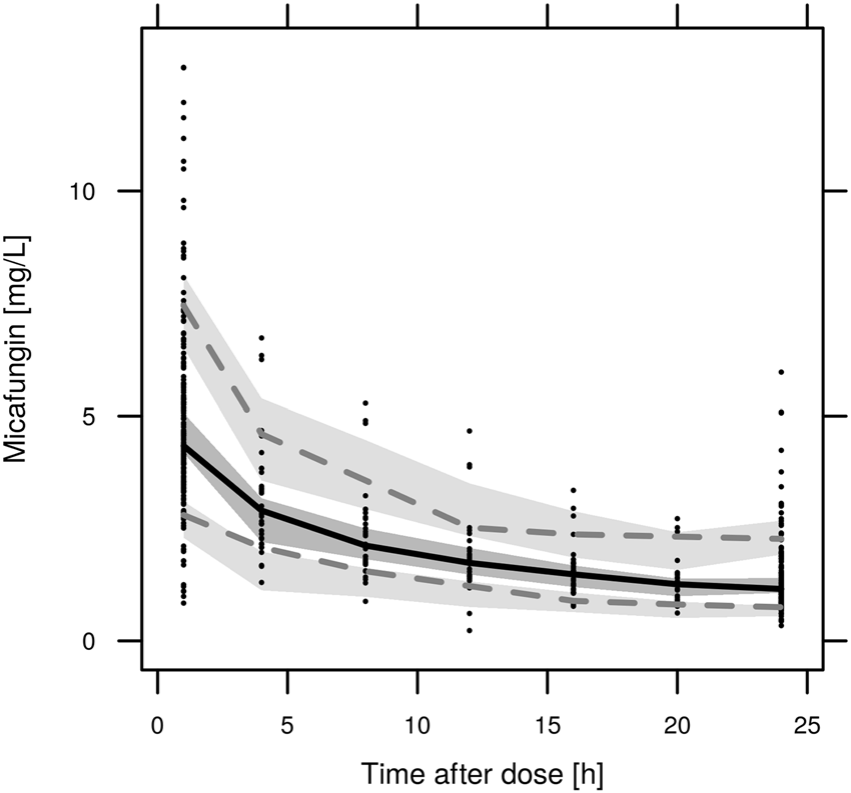
Visual predictive check for the final Micafungin model; black dots, observed values; grey areas, 90% confidence interval predicted percentiles; lines, median and 5th plus 95th percentiles for the observed values.

**Figure 6:**
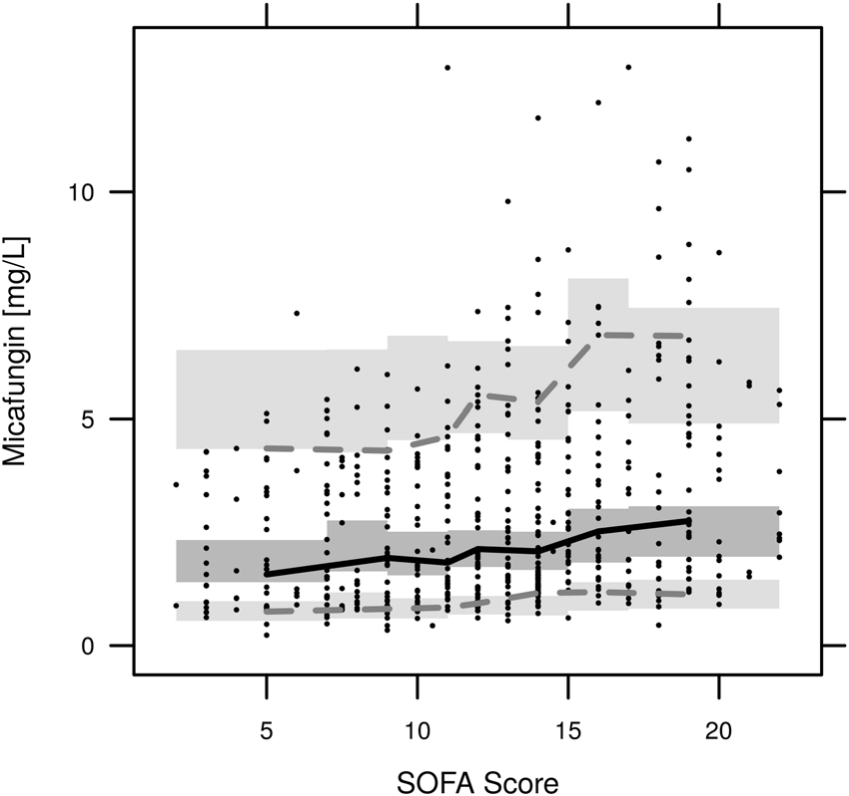
Visual predictive check for predictivity of the final micafungin model across the SOFA score range; black dots, observed values; grey areas, 90% confidence interval predicted percentiles; lines, median and 5th plus 95th percentiles for the observed values.

Internal model validation performed by GOF (goodness of fit) plots showed an even distribution of residuals over TAD (time after dose) and PRED (population model predictions) (Fig. 4). A VPC (visual predictive check) was created against time after dose and against the SOFA score to investigate the appropriateness of the model over the whole time and SOFA range, as shown in Figs 5, 6. In this VPC, the observed percentiles and median matched the corresponding simulated confidence intervals, indicating an adequate predictive performance of the final model.

### Pharmacodynamic target evaluation

**Figure 7:**
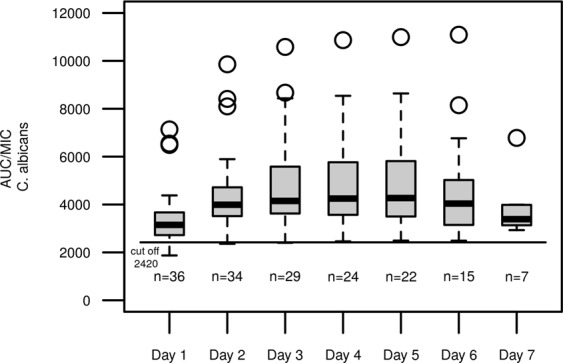
AUC/MIC ratio for micafungin and *C*. *albicans* in the observed study population; Each box represents 24 h of therapy, numbers below boxes show the number of patients enrolled in the study at the respective time point, solid line represents the EUCAST cut off value for antifungal activity for *C*. *albicans*.

**Figure 8:**
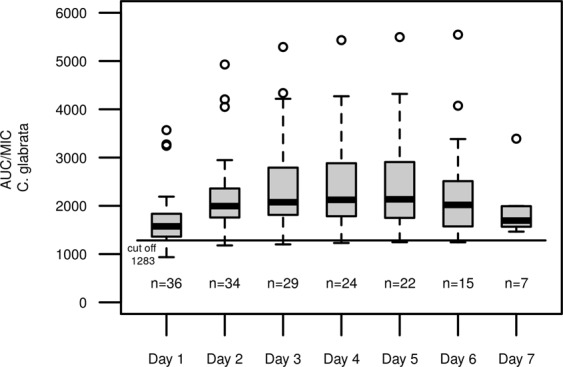
AUC/MIC ratio for micafungin and *C*. *glabrata* in the observed study population; Each box represents 24 h of therapy, numbers below boxes show the number of patients enrolled in the study at the respective time point, solid line represents the EUCAST cut off value for antifungal activity for *C*. *glabrata*.

**Figure 9:**
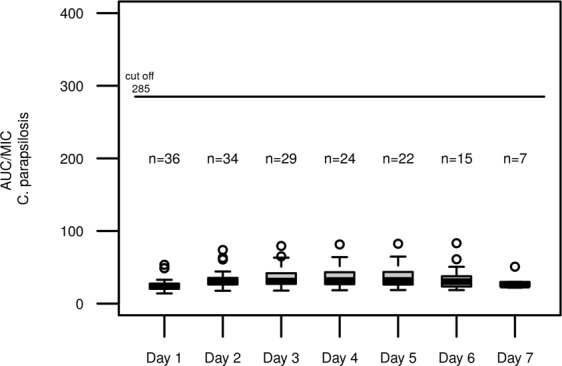
AUC/MIC ratio for micafungin and *C*. *parapsilosis* in the observed study population; Each box represents 24 h of therapy, numbers below boxes show the number of patients enrolled in the study at the respective time point, solid line represents the EUCAST cut off value for antifungal activity for *C*. *parapsilosis*.

In a post-hoc estimation step, the AUC was determined for each patient on each day of treatment. The resulting AUC/MIC ratios for different candida species are shown in Figs 7–9. When compared to common *Candida* species breakpoints, the AUC/MIC ratios show adequate therapy intensities for both *C*. *albicans* (Fig. 7) and *C*. *glabrata* (Fig. 8), whereas a fixed dose of 100 mg micafungin does not seem suitable for the less sensitive *C*. *parapsilosis* strains (Fig. 9).

## Discussion

Since micafungin was licensed for clinical use in 2008, various parametric and non-parametric PopPK models have been published. In accordance with our findings, they all consist of two compartments with linear elimination^12,15,17–19^.

The reported PopPk model well describes the pharmacokinetics of micafungin in this critically ill population. The main objective of this study was to further investigate the influence of different types of renal replacement therapy on micafungin kinetics. In our analysis, renal replacement therapy did not alter the pharmacokinetics of micafungin. Neither continuous dialysis (CVVHDF), nor intermittent modes of dialysis (SLEDD) caused appreciable removal of micafungin. This finding is in accordance with the known elimination routes of micafungin and adds to the recently published observations of Vossen *et al*.^20^.

Rather than the status of kidney failure or renal replacement therapy, the overall severity of illness as reflected by the SOFA score has an influence on elimination and distribution of micafungin. In patients suffering from liver dysfunction with bilirubin levels above 4 mg/dl as part of multi organ dysfunction, Micafungin clearance decreased. This finding is in accordance with the biliary excretion of micafungin, which will be affected in situations of liver dysfunction.

Taken together, the results of our analysis are in keeping with other published findings examining pharmacokinetics of micafungin in critically ill patients^17,19,21,22^. However, the covariate model differs slightly from previous findings.

Martial *et al*.^19^ tested, but could not confirm an influence by the covariates albumin, Child Pugh Score, SOFA Score or renal replacement therapy in a parametric PopPk analysis. Jullien *et al*.^17^ reported a categorical influence of the SOFA score as well as albumin levels on the micafungin clearance, explored by a parametric PopPk analysis. These dissimilar findings might be explained by differences in the time points of blood sampling. In our study, patient samples were collected for up to seven days, if therapy with micafungin was continued. This allowed a valid analysis of time-dependency of different factors and their influence on pharmacokinetics, that have not been covered in previous analyses.

In this cohort of critically ill patients with suspected or proven infection with *Candida ssp*., once daily dosage of 100 mg micafungin resulted in adequate plasma levels of the drug in all patients, when assuming the EUCAST-breakpoints for *C*.*albicans* and *C*. *glabrata* (Figs 7, 8). In infections with *C*. *parapsilosis*, our analysis revealed inadequate exposure with once daily dosing due to higher breakpoints (Fig. 9). This finding corroborates previous reports of questionable efficacy of micafungin in this particular fungus^23^. We conclude that alternative substances might be a preferable choice, for example azole antifungals or preparations of Amphotericin B.

As the EUCAST-breakpoints are slightly lower than their CLSI counterparts, our analysis differs from other studies that aim to attain numerically higher pharmacokinetic targets^21,22^. As the clinical relevance of this difference in targets is unknown, we feel that a widespread use of increased doses for non-parapsilosis *Candida* species is not warranted at the moment^22^.

Nevertheless, the recommended dosing regimen of once daily application of 100 mg micafungin can be considered adequate for the majority of critically ill patients with suspected or proven candidiasis. Renal replacement therapy does not have an influence on the disposition of micafungin and changes in dosing are not warranted in this collective. Therefore, our study adds to the body of evidence demonstrating the adequacy of a comparatively easy dosing scheme for micafungin without the necessity of dynamic adjustments in a wide range of patients with critical illness. However, if infections with *C*. *parapsilosis* are detected, target attainment is more difficult than in cases of infection with *C*. *albicans* or *C*. *glabrata*. If a switch to alternative substances is not feasible in these cases, micafungin therapy would benefit from therapeutic drug monitoring and resistance testing in order to reach sufficient PK/PD-targets.

## Methods

### Study protocol

This is a non-interventional study conducted in critically ill adult patients treated in the intensive care units of the Department of Anaesthesiology, Intensive Care Medicine and Pain Therapy of the University Hospital Muenster in Germany. The collection of data and blood samples was approved by the ethics committee of the University of Muenster (Study Code 06-AnIt-11). All procedures performed in in this study were in accordance with the ethical standards of the institutional and/or national research committee and with the 1964 Helsinki declaration and its later amendments or comparable ethical standards. Written informed consent was obtained from all patients or their representatives.

The study included 36 adult patients. Patient demographics and other clinical parameters were collected from the electronic patient charts of the intensive care unit. All patients received 100 mg micafungin intravenously once daily either as empiric or targeted therapy of suspected or proven infection. The decision to begin or end therapy was made by the physicians in charge of the intensive care units irrespective of the pharmacologic study.

Blood samples were collected from indwelling arterial catheters for analysis of micafungin concentrations. On day 1, plasma micafungin concentration was determined at 1, 4, 8, 12, 16 and 20 hours post application and as a trough level immediately before the next dose of micafungin after 24 hours. From day two of therapy, peak levels one hour after infusion and trough levels were collected.

### Blood sampling

Blood samples were exclusively taken from arterial lines into ethylenediaminetetraacetic acid (EDTA)-containing sample vials tubes (S-Monovette® 2.7 ml K3E, Sarstedt, Nümbrecht, Germany). Samples were immediately transported to the in-house laboratory and plasma was separated by centrifugation at 1600 x g (10 min) and kept and frozen at −80 °C until analysis.

### Sample preparation and analysis of micafungin

Micafungin in human plasma was analyzed with reversed-phase high-performance liquid chromatography. 200 µl serum, 20 µl internal standard solution (anidulafungin 1 mg/ml in water), 20 µl 30% ZnSO_4_ x7H_2_O (w/v) in water and 700 µl methanol were vortex-mixed (5 sec) followed by centrifugation at 800 g for 3 min. 50 µl of the supernatant was injected on a 125 liquid chromatograph (Beckman Coulter GmbH, Krefeld, Germany) interfaced with a model 168 diode array detector. Chromatographic conditions: mobile phase: acetonitrile/50 mM ammonium acetate buffer pH 4.6, 50:50 (v/v); flow rate 1 ml/min at 45 °C; absorbance detection 271 nm; column: XTerra RP18, 3.5 µm, 150 × 4.6 mm (Waters GmbH, Eschborn, Germany).

The method was validated according to the guidelines for bioanalytical methods published by the European Medicines Agency^24^. The recoveries using this precipitation procedure were 99 ± 3.3% and 96.2 ± 5.8% for 5 and 200 µg/mL micafungin in serum, respectively. The lower limit of quantitation (LLOQ), the limit of detection (LOD) and the linear range for micafungin analysis were 0.1 µg/ml, 0.02 µg/ml, and 0.1–200 µg/ml, respectively. The intra- and inter-day precision of the assay expressed as a coefficient of variation (CV%) ranged from 3.7 to 7.2%.

### Population pharmacokinetic modelling

Data pre-analysis and graphical output was created in R (version 3.4.0; R foundation for statistical computing, Vienna, Austria) with additional use of the Xpose package (version 4.5.3).

Model building was performed using NONMEM (Version 7.3; ICON Development Solutions, Ellicott City, MD) with the ADVAN 6 subroutine and the FOCE + I estimation method. Visual predictive checks (pcVPC) and stepwise covariate model building (SCM) were performed with the PsN module (Version 4.6.0)^25^. Graphical output was created in R (version 3.4.0; R foundation for statistical computing, Vienna, Austria) with additional use of the Xpose package (version 4.5.3)^26^.

The basic structural model was tested with the following compositions: One to three compartments with linear clearance, nonlinear *(Michaelis Menten)* clearance and a combination of both were tested.

There was no covariate inclusion before the basic structural model was defined.

For nested models, the difference in the objective function value (OFV) was considered the best parameter to quantify model improvement. A drop in OFV of 3.84 and 6.63, corresponding to a 5% and 1% level of significance respectively, was considered an adequate model improvement, when a single parameter was added. Non-hierarchical models were compared using the *Akaike* information criterion (AIC) in combination with goodness of fit (GOF) plots^27,28^.

All covariates were collected as time-varying covariates, if feasible.

Potential continuous covariates included demographics such as weight, age and BMI. Additionally, albumin and serum-protein were documented to depict changes in protein binding. Renal function was documented for non-RRT-patients by creatinine clearance (CrCl) and creatinine values. Hepatic function was characterized through documentation of alanine aminotransferase (ALT), aspartate aminotransferase (AST), butyrylcholinesterase (BCHE) and bilirubin. As general scores, Acute Physiology and Chronic Health Evaluation II (APACHE II) was recorded on admission to the ICU and both Sepsis-related organ failure assessment score (SOFA) and Simplified Acute Physiology Score (SAPS2) scores were documented each day in the ICU.

As categorical covariate, sex was tested. Covariate influence was tested on central and peripheral volumes of distribution, and clearance. Covariate testing was performed using stepwise covariate modelling (SCM) in PsN with a 5% forward inclusion and 1% backward elimination criterion^29^.

Model performance was evaluated by creating GOF plots including individual predictions (IPRED) and population predictions (PRED) versus the observed values, and plots for conditional weighted residuals (CWRES) versus time, time after dose (TAD) and the population model predictions (PRED). Visual predictive checks (VPCs) were created to test the predictive performance of the model.

### Pharmacodynamic target evaluation

The *European Committee on Antimicrobial Susceptibility Testing* (EUCAST) established the guideline for micafungin and *Candida ssp*. with species related clinical breakpoints for non-resistant strains^10^. These breakpoints were used as surrogate for the minimal inhibitory concentration (MIC) for *C*. *albicans*, *C*. *glabrata* and *C*. *parapsilosis*. Proposed MICs are 0.016 g/L, 0.03 g/L and 2 g/L, respectively.

Additionally, EUCAST suggests the following AUC/MIC targets to reach stasis: *C*. *albicans* (2420), *C*. *glabrata* (1283), *C*. *parapsilosis* (285).

Using the previously established population pharmacokinetic model, the AUC for each patient on each day of therapy was determined. In a post-hoc estimation step, micafungin concentrations in the central compartment were used to interpolate for AUC 0-24 h on each day of treatment and the AUC/MIC ratio was calculated and compared to the guideline suggestions.

## Data Availability

The datasets generated and analyzed during the current study are available from the corresponding author on reasonable request.
